# The Interleukins Orchestrate Mucosal Immune Responses to *Salmonella* Infection in the Intestine

**DOI:** 10.3390/cells10123492

**Published:** 2021-12-10

**Authors:** Fu-Chen Huang

**Affiliations:** Department of Pediatrics, Kaohsiung Chang Gung Memorial Hospital and Chang Gung University College of Medicine, Kaohsiung 833, Taiwan; huang817@cgmh.org.tw; Tel.: +886-7-7317123 (ext. 8724)

**Keywords:** interleukin, *Salmonella*, immune response, intestine, mucosa

## Abstract

Salmonella infection remains one of the major public health problems in the world, with increasing resistance to antibiotics. The resolution is to explore the pathogenesis of the infection and search for alternative therapy other than antibiotics. Immune responses to Salmonella infection include innate and adaptive immunity. Flagellin or muramyl dipeptide from Salmonella, recognized by extracellular Toll-like receptors and intracellular nucleotide-binding oligomerization domain2, respectively, induce innate immunity involving intestinal epithelial cells, neutrophils, macrophages, dendric cells and lymphocytes, including natural killer (NK) and natural killer T (NKT) cells. The cytokines, mostly interleukins, produced by the cells involved in innate immunity, stimulate adaptive immunity involving T and B cells. The mucosal epithelium responds to intestinal pathogens through its secretion of inflammatory cytokines, chemokines, and antimicrobial peptides. Chemokines, such as IL-8 and IL-17, recruit neutrophils into the cecal mucosa to defend against the invasion of *Salmonella*, but induce excessive inflammation contributing to colitis. Some of the interleukins have anti-inflammatory effects, such as IL-10, while others have pro-inflammatory effects, such as IL-1β, IL-12/IL-23, IL-15, IL-18, and IL-22. Furthermore, some interleukins, such as IL-6 and IL-27, exhibit both pro- and anti-inflammatory functions and anti-microbial defenses. The majority of interleukins secreted by macrophages and lymphocytes contributes antimicrobial defense or protective effects, but IL-8 and IL-10 may promote systemic *Salmonella* infection. In this article, we review the interleukins involved in Salmonella infection in the literature.

## 1. Introduction

*Salmonella* infection remains one of the major public health problems in the world, with increasing resistance to antibiotics. Non-typhoidal *Salmonella* (NTS) usually causes self-limiting diarrhea in immunocompetent hosts, but may develop into sepsis or complications in immunocompromised hosts.

Immune responses to *Salmonella* infection include innate and adaptive immunity. The different stages of *Salmonella* infection are reflected in the innate and acquired immunity, orchestrated by a variety of immune cells to defend against this bacterium, having a different importance during distinct infection stages. The innate immune system can restrict the replication of *Salmonella* to a certain degree, but acquired immunity is essential for the effective control and eradication of bacteria.

Besides intestinal epithelial cells which form a physical barrier and produce inflammatory cytokines, chemokines, and antimicrobial peptides [1,2], a variety of immune cells accomplish the innate immunity against *Salmonella* infection, including dendritic cells, neutrophils, macrophages, natural killer (NK), and γδ T cells. Phagocytes, central to the control of *Salmonella* infection during the initial stages of *Salmonella* infection, are recruited and activated by the inflammation of the infected tissues and large amounts of IFN-γ produced by a variety of cells, with NK cells being an important source [3]. Interleukin (IL)-8 recruits neutrophils from the circulation system into the infected tissue to defend against the invasion of *Salmonella* [4]. However, the accumulation of neutrophils gives rise to characteristic pathological changes of colitis. Macrophages appear to be crucial for protective immunity against intracellular *Salmonella* by phagocytosis of the bacteria and, along with dendritic cells, are major sources of many interleukins [5,6]. Moreover, dendritic cells play an important bridge between innate and acquired immunity via interleukins [7]. T cells are important for the control of *S.* typhimurium infection and CD4+ T cells in particular, but also CD8+ T cells and perhaps γδ T cells are involved [5]. Humoral immunity plays critical role(s) in the response to *Salmonella* infection, especially at the late phase [8]. Among intracellular bacteria, B cells play a notable role in resistance to *Salmonella*.

Infection leads to Toll-like receptors or intracellular nucleotide-binding oligomerization domain activation, the production of inflammatory interleukins such as IL-1α/β, IL-6, IL-8, IL-10, IL12/23, IL-15, IL-17A, IL-18, IL-25, IL-27, TNF-α, chemokine (C-C motif) ligand 2 (CCL2), IFN-γ, and neutrophil and macrophage recruitment. The plasma pro-inflammatory versus anti-inflammatory cytokine profile in patients with severe sepsis has been demonstrated to predict mortality [9,10,11,12,13,14]. In this article, we review the interleukins orchestrate intestinal mucosa responses to *Salmonella* infection in the literature (Table 1 and Table 2).

## 2. IL-1

Interleukin (IL)-1α and IL-1β are equally potent inflammatory cytokines but reveal highly dissimilar functions and biogenesis. IL-1α is constitutively expressed in many cell types at a steady state, and can be induced in response to cell stress, injury, infection, or pro-inflammatory stimuli [64]. IL-1α can function as a plasma membrane-bound cytokine. The exposure of cells to bacterial infection stimulates the intracellular expression of IL-1α as well as resulting in membrane IL-1α expression [65,66,67]. Although the biogenesis of IL-1α and its distinctive role in the inflammatory process remain poorly defined, recombinant murine TNF-α and IL-1α can protect mice from lethal bacterial infections [68]. The resistance of mice to a lethal dose of *S. typhimurium* could be enhanced if the mice were pretreated with IL-1α [15]. IL-1β is a key mediator of the inflammatory response and plays a major role on the pathogenesis of inflammatory bowel disease (IBD), consistent with the finding that IL-1β is up-regulated in IBD patients [48] and IBD colonic macrophages release mature IL-1β on exposure to lipopolysaccharide (LPS) [47]. The IL-1β-induced increase in the intestinal epithelial tight junction permeability may contribute to intestinal inflammation. Autophagy protein Atg16L1 polymorphisms in Crohn disease exhibit an excessive production of IL-1β and overwhelming inflammation in the colon [69,70]. Active vitamin D might enhance autophagy and decrease the IL-1β response to defend against *Salmonella* invasiveness and prevent the host from detrimental inflammation [2]. In vivo, active vitamin D3 attenuates the severity of *Salmonella* colitis in mice by decreasing IL-1β expression [50]. Furthermore, specific blockade of IL-1 receptors reduces the inflammatory responses in rabbit immune complex colitis [49]

## 3. IL-6

Interleukin (IL)-6 produced by enterocytes has anti-inflammatory and cell-protective effects in intestinal mucosa and enterocytes. It may counteract some of the injurious effects of sepsis and endotoxemia [17,71]. IL-6 has been reported to be a mediator of the epithelial barrier protection [72] and endogenous IL-6 plays an essential, non-redundant role in limiting intestinal injury [16]. *Salmonella*-induced intense inflammation causes the breakdown of the intestinal epithelial barrier, translocation of bacteria, and absorption of endotoxins into the circulation [73] and, consequently, bacteremia as well as endotoxemia. The probiotic *Lactobacillus paracasei* may exert some of their beneficial effects by enhancing IL-6 production in enterocytes subjected to an inflammatory stimulus [18]. PJ-34, a potent poly (ADP-ribose) polymerase-1 (PARP-1) inhibitor, may exert defense on intestinal epithelial cells (IECs) against invasive *Salmonella* infection by up-regulating IL-6 production through the ERK and NF-κB signal pathway [19].

## 4. IL-8

Current data indicate that IECs orchestrate mucosal innate immunity through their production of inflammatory cytokines, chemokines, and antimicrobial peptides [74,75]. Chemokines, such as IL-8, recruits neutrophils into cecal mucosa to defend against the invasion of *Salmonella* [20,21,22]. However, the accumulation of neutrophils gives rise to colitis [76] and sepsis [51]. Toll-like receptor 5 (TLR5) and intracellular nucleotide-binding oligomerization domain 2 (NOD2) are two important pattern recognition receptors involved in innate immunity to invading pathogens. Flagellin, a ligand for TLR5, is a dominant pro-inflammatory determinant in IECs infected by *Salmonella*. The cooperation of flagellin and muramyl dipeptide, a NOD2 agonist, in IECs synergistically upregulates inflammatory IL-8 response to *Salmonella* infection [23]. Intracellular *Salmonella* infection in both macrophages and IECs induces cholesterol accumulation in the *Salmonella*-containing vesicles (SCVs) [77] within which the virulent bacteria survive and replicate [78,79]. Plasma membrane cholesterol supports the survival of *Salmonella* in IECs through the PI3K-dependent anti-IL-8 pathway [24]. Contrasting to membrane cholesterol, sphingolipids act on epithelial defense against the invasive pathogen by enhancing the NOD2-mediated human beta-defensin 2 (hBD-2) response [80]. The probiotic *Lactobacillus plantarum* Lp62 inhibited IL-8 production by *Salmonella* Typhi-stimulated IECs and prevented the adhesion of pathogens to the cells [57]. The treatment of probiotics before and after infection having different effects on the *Salmonella*-induced IL-8 response in IECs suggests the critical timing of probiotic supplementation [25]. Active vitamin D modulates the pro-inflammatory IL-8 response in *Salmonella*-infected IECs to prevent the host from detrimental extreme inflammation [1].

## 5. IL-10

IL-10 is a powerful anti-inflammatory Th2 cytokine with a broad range of target cell types, primarily of the innate cells, such as dendric cells (DCs), macrophages, neutrophils, B cells, and T cells. Mice deficient in IL-10 spontaneously develop colitis resembling IBD. During *Clostridium difficile* infection, IL-10 is required to avoid an excessive immune reaction and prevent the host from more severe acute colitis [81]. IL-10 can dampen the secretion of pro-inflammatory cytokines, such as IL-12 [26] and prevents tissue damage [82] by acting on antigen-presenting cells. Furthermore, IL-10 facilities the regulatory T (Treg) cell-mediated suppression of T helper 17 (Th17)-driven colitis in mice [27]. On the other hand, *S.* Typhimurium can infect and persist in B cells [83], which provide additional signals to transform T cells to Treg cells. IL-10 produced by T and B cells promotes systemic *Salmonella enterica* serovar Typhimurium infection in mice [52]. The in vivo administration of the anti-IL-10 monoclonal antibody significantly enhanced host resistance at the early stage of *Salmonella* infection by accelerating macrophage functions and, consequently, the activation of γδT cells and enhanced levels of monokine mRNA, including IL-lα, tumor necrosis factor-α (TNF-α), and IL-12 [53].

## 6. IL-12

IL-12, similar to IL-23, is a pro-inflammatory cytokine produced by activated DCs, macrophages in response to microbial pathogens [84,85], and stimulates natural killer (NK), T, and natural killer T (NKT) cells to produce IFN-γ, which, in turn, aid in the elimination of intramacrophage pathogens, including *S.* Typhimurium [30,86]. These two cytokines must be considered together, because both share a common p40 subunit and their biology is closely interlocked [7]. IL-12 and IL-23 can also induce TNF-α and GM-CSF production in T cells via IFN-γ-independent signal transduction and bactericidal mechanisms [28]. In a human study, a high incidence of invasive *Salmonella* diseases in patients with IL-12/IL-23-INF-γ-axis deficiency highlights the importance of IL-12/IL-23- INF-γ-axis for immunity against *Salmonella* in humans [29]. Clinicians should consider an underlying IL-12/IL-23-INF-γ-axis deficiency in patients with recurrent, extraintestinal, and invasive *Salmonella* diseases, which usually require extensive treatment. Recombinant gamma interferon enhances the internalization and early intracellular killing of *Salmonella enterica* serovar Typhimurium by human macrophages [30], which release more IL-12 and less IL-10. Ustekinumab, the monoclonal antibody targeting the shared p40 subunit, has been approved for Crohn’s disease (CD) and has demonstrated promising results in the treatment of ulcerative colitis [54].

## 7. IL-15

Interleukin-15 (IL-15) is a cytokine that resembles IL-2 in its biological activities [31], stimulating macrophages, NK cells, T cells, and B cells to secrete cytokines, and has pro-inflammatory properties by itself [55]. It is upregulated under the conditions of tissue destruction and during infection [87,88]. IL-15 was reported to be involved in protection against bacterial infection mediated by NK and γδ T cells [31]. IL-15 overexpression promotes intestinal dysbiosis with a decrease in luminal butyrate-producing bacteria, lowers butyrate levels, and is associated with an increased susceptibility to colitis [56] and impacts on the pathogenesis of intestinal inflammatory diseases, such as celiac disease and IBD. Endogenous IL-15 had an important function in early protection against infection with an avirulent strain of *S. choleraesuis* through the activation of NK cells and IFN- γ production [32].

## 8. IL-17

Interleukin-17 (IL-17), a cytokine produced by Th17 cells, recruits neutrophils and plays a crucial role in host defense against extracellular bacteria [33]. Additionally, IL-17 helps to orchestrate mucosal inflammation by inducing the production of neutrophil chemoattractants (e.g., IL-8, CCL20, Lipocalin-2, and iNOS) in the intestines [41]. In IBD patients, the increased expression of IL-17 was observed in the intestinal mucosa [89]. In the mouse colitis model of *S.* Typhimurium infection, IL-23 orchestrates mucosal responses to release IL-17 [41] that contribute specifically to neutrophil recruitment into the cecal mucosa to prevent *Salmonella* dissemination [34,35]. iNKT cells play a protective role against *Salmonella* enterocolitis by downregulating IL-17-producing γδT cells [58]. The probiotic *Lactobacillus plantarum* ZS2058 significantly reduced the pathogenicity of *Salmonella* colitis by promoting the colon IL23/IL-22 axis in the mouse [36]. *Lactobacillus plantarum* Lp62 was able to suppress IL-17, IL1-β, and TNF-α production in LPS-stimulated J774 macrophages [57].

## 9. IL-18

IL-18 is a cytokine that binds to a specific receptor expressed on various types of cells and has pleiotropic functions [37,59]. Its capability to promote INF-γ production by T cells and NK cells leads to pro-inflammatory activity. Colon epithelial cells constitutively produce IL-18, which increases upon NLRP6 inflammasome and in turn activates the epithelial cells to produce antimicrobial peptides to maintain microbiome homeostasis [38]. Moreover, the binding of TLR5 to its ligand flagellin not only results in a NF-κB-mediated pro-inflammatory cytokine responses, including IL-8, TNFα, and the matrix metalloprotease (MMP)-9, but induces IL-18 secretion and Th1-like cytokine responses in human peripheral blood mononuclear cells (PBMC) [37]. *Salmonella* pathogenicity islands (SPI)-1 effector secretion leads to NF-κB signaling and caspase-1-mediated IL-1β/IL-18 activation [60].

## 10. IL-22

The major functions of IL-22 in the intestine are the inflammatory response, enhanced antimicrobial activity, the maintenance of the mucosal barrier, resistance to colonization of opportunistic pathogens, enhancement of epithelial regeneration, and wound healing [40,90]. IL-22 can restrict the growth of *M. tuberculosis* intracellularly in macrophages by enhancing phagosomal fusion. IL-22 can also orchestrate mucosal inflammation by inducing the production of neutrophil chemoattractants (e.g., IL-8, CCL20, Lipocalin-2, and iNOS) in the intestines [41]. IL-22 promotes the intracellular fusion of SCVs with lysosomes, leading to the phagolysosomal killing of *S.* Typhimurium in human epithelial cells [39]. *Salmonella*-induced IL-22 production can suppress the growth of commensal *Enterobacteriaceae*, the closest competitors for *Salmonella,* in the inflamed gut, thereby enhancing the *Salmonella* colonization of mucosal layers [91]. Furthermore, the ability of IL-22 to heal intestinal inflammation and promote epithelial repair from acute injury [40] highlights IL-22 as a promising target for future IBD therapy.

## 11. IL-23

Interleukin-23 (IL-23) is a member of the IL-12 family of cytokines with pro-inflammatory properties. Its ability to potently enhance the expansion of Th17 cells indicates the responsibility for many of the inflammatory autoimmune responses. IL-23 is a key participant in the central regulation of the cellular mechanisms involved in inflammation [61]. IL-23 is produced by various immune cells such as dendritic cells, monocytes, as well as type 1 macrophages (Mϕ1) upon Toll-like receptor signaling [92] in response to the binding of pathogens. In addition, neutrophils have been identified as a potential source of IL-23 production [93]. Recent studies have identified an increased production of IL-23 in various mouse models of colitis and IBD patients (review in [63]). IL-23 is able to accelerate the proliferation of both murine and human memory T cells producing Th17 cytokines such as IL-17A, IL-17F, and IL-22 [62]. IL-23 is known to induce IFN-γ production and is associated with host innate immunity against *Salmonella*. The IL-23/IL-22 axis during innate immunity against *Salmonella* may contribute to protection against *Salmonella* infection by several ways, such as IL-22-regulated expression of anti-microbial peptides and acute phase proteins and IL-17A-dependent neutrophil recruitment [42]. IL-23 deficient mice were unable to express IL-17A during *S.* Typhimurium-induced colitis [41]. Infection with *Salmonella* can induce IL-23, IL-18, and IL-1β, but not IL-12, production in monocytes and type 1 pro-inflammatory macrophages [94].

## 12. IL-27

Interleukin (IL)-27, one of heterodimeric cytokines that belongs to the IL-12 family, has fundamental roles in innate and adaptive immune regulation [95]. It is produced in myeloid cells in response to bacterial infection and has both anti- and pro-inflammatory functions [95,96]. Accordingly, the role of IL-27 in human and experimental mouse colitis is controversial. IL-27 enhanced TLR4 or TLR5 expression in human monocytes and macrophages, induced greater LPS/flagellin-mediated signaling, and significantly enhanced pro-inflammatory cytokines IL-12p40, TNF-α, and IL-6 production in *S. typhimurium*-infected cells [43,44,46]. These findings support the role for IL-27 in anti-microbial defenses by altering the expression of innate immune sensors such as TLR4 or TLR5 [45].

## 13. Conclusions

*Salmonella* infection remains one of the major public health problems in the world, with increasing resistance to antibiotics. The resolution is to look for alternative therapy other than antibiotics based on the host immune reaction to infection. Interleukins play a crucial role in orchestrating mucosal immune responses to *Salmonella* infection in the intestine. How to take advantage of is knowledge and exploit effective reagents to treat *Salmonella* infection would be the essential next-generation issue.

In recent years, our group has contributed much effort to exploit the biotherapy that could prevent or treat *Salmonellosis*. PJ-34, a PARP-1 inhibitor, may exert its protective effect on intestinal epithelial cells against invasive *Salmonella* infection by up-regulating IL-6 production, which has anti-inflammatory and cell-protective effects [19], and counteracts some of the injurious effects of sepsis and endotoxemia.

*Salmonella*-induced plasma membrane cholesterol accumulation in SCVs [24] may, subsequently, protect intestinal epithelial cells from apoptosis and produce an anti-inflammatory signal, both of which may contribute to the establishment of a *Salmonella* infection in cells. Contrasting to the utilization of membrane cholesterol on the maintenance of *Salmonella*-containing vacuoles and anti-inflammatory responses, sphingolipids act on the epithelial defense against the invasive pathogen [80]. Simvastatin or Fluvastatin, cholesterol-lowering statins, can suppress the pro-inflammatory IL-8 response in *Salmonella*-infected IECs to prevent the detrimental effects of overwhelming inflammation in the host [97].

Our recent in vitro and in vivo studies observed that active vitamin D prevented the host from the detrimental effects of overwhelming inflammation by downregulating pro-inflammatory responses (IL-6, TNF-α, IL-8, and IL-1β) [1,2,25] in *Salmonella*-infected IECs and decreased the severity of colitis in mice. The different regulation of probiotics on *Salmonella*-induced IL-8 responses in Caco-2 cells according to the administered timing supports a rationale for the therapeutic use of probiotics in the treatment of *Salmonella* colitis and inflammatory bowel disease [25]. Furthermore, we observed the synergistic effects of probiotics or postbiotics and active vitamin D on anti-inflammatory responses (IL-6, TNF-α, IL-8, and IL-1β) in *Salmonella* colitis mice [98,99].

It is mandatory to elucidate the pathogenesis of *Salmonella* infection for the design of intervention strategies that might reduce the use of antimicrobial agents and decrease the incidence of multidrug-resistant *Salmonellosis*. All these novel findings and thoughtful explorations of health knowledge could be applied to perform clinical trials and preventive medicine for the better lives of future generations.

## Figures and Tables

**Table 1:** The interleukins orchestrate mucosal immune responses to defense against *Salmonella* infection in the intestine.

Interleukin	Biologic Functions	Experiments	Intervention and Effects	Clinical Applications	Ref.
IL-1α	Function as a plasma membrane cytokine involved in the inflammation and protection from bacterial infections though its role remains poorly defined	Mice	IL-1α-enhanced resistance of mice to *S.* Typhimurium infection	*Salmonella*	[15]
IL-6	Anti-inflammation Mediator of epithelial barrier protection Protection from sepsis and endotoxemia	IECs	PJ-34 up-regulates IL-6 production in *Salmonella*-infected IECs		[16,17,18,19]
		Enterocytes	Probiotic (*L. paracasei*) potentiates IL-6 production in IL-1beta-treated Caco-2 cells		
IL-8	Recruits neutrophils to defense against the invasion of *Salmonella*	IECs	Flagellin and MDP synergistically enhance IL-8	*Salmonella* colitis IBD	[20,21,22,23,24,25]
			2. Plasma membrane cholesterol supports the survival of *Salmonella* in IECs through anti-IL-8 pathways		
			3. Probiotics enhance IL-8 expression		
IL-10	Anti-inflammation Th2 cytokine Inhibit the development of Th1-type immune responses Reduce NK cell responses Prevent the differentiation of naïve T cells into effector cytotoxic T cells Dampen the secretion of pro-inflammatory cytokines, such as IL-12 Induce Treg cell proliferation Suppression of T helper 17 (TH17)-driven colitis	Mice	IL-10-deficient mice develop colitis	*Salmonella* sepsis	[26,27]
IL-12	IL-12/IL-23 component acts on NK and T cells and NKT cells to induce IFN-γ-dependent or IFN-γ-independent immunity against intracellular *Salmonella* infection A Key cytokine for immunity against invasive *Salmonella* in humans	Humans	IL-12 enhances internalization and early intracellular killing of *Salmonella enterica* Serovar Typhimurium by human macrophages	Recurrent, extraintestinal and invasive *Salmonella* diseases	[28,29,30]
IL-15	Stimulating macrophages, NK cells, T cells, and B cells to proliferate, secrete cytokines, and/or produce antibody Protection against bacterial infection	Mice	Endogenous IL-15 functions as early protection against infection with an avirulent strain of *S. choleraesuis* through activation of NK cells and IFN- γ production	*Salmonella* infection	[31,32]
IL-17	A cytokine involved in neutrophil recruitment to defend against extracellular bacteria	Mice	Probiotic *Lactobacillus plantarum* ZS2058 significantly reduced the pathogenicity of *Salmonella* colitis by promoting the IL23/IL-22 axis in the mouse ileum	*Salmonella* colitis	[33,34,35,36]
IL-18	Promotes IFN-γ production by T cells and NK cells thereby shaping immunity towards a Th1-like phenotype Activates the colon epithelial cells to produce antimicrobial peptides to maintain microbiome homeostasis		*Salmonella* pathogenicity islands (SPI)-1 effector secretion leads to NF-kB signaling and caspase-1-mediated IL-1β/IL-18 activation		[37,38]
IL-22	Inflammatory responses Maintenance of intestine mucosal barrier Enhanced antimicrobial activity, and mucosal healing Resistant to intestinal colonization of opportunistic pathogens	Human epithelial cells	IL-22 promotes intracellular fusion of SCVs with lysosomes leading to phagolysosomal killing of *S.* Typhimurium in human epithelial cells	*Salmonella* colitis IBD	[39,40]
		Mice	IL-22 is able to heal intestinal inflammation and promote epithelial repair from acute injury		
IL-23	A member of the IL-12 family of cytokines with pro-inflammatory properties IL-23 induce IFN-γ and IL-22 production and are associated with host innate immunity against *Salmonella*	Mice	Mice deficient for IL-23 is associated with *S.* Typhimurium colitis	IBD *Salmonella* colitis	[41,42]
IL-27	Has fundamental roles in innate and adaptive immune regulation Has both anti- and pro-inflammatory functions Enhance TLR4 or TLR5 expression in human monocytes and macrophages, to cooperate for optimal anti-bacterial responses	Monocytes Macrophages	IL-27-enhanced TLR4 or TLR5 expression in human monocytes and macrophages, induced greater LPS/flagellin-mediated signaling, and significantly enhanced pro-inflammatory cytokines IL-12p40, TNF-α, and IL-6 production in *S. typhimurium* infected cells	*Salmonella* infection	[43,44,45,46]

Abbreviations: IECs, intestine epithelial cells; IBD, inflammatory bowel disease; MDP, muramyl dipeptide; NK, natural killer; Treg: regulatory T cells; NKT, natural killer T; INF, interferon; TNF, tumor necrosis factor; LPS, lipopolysaccharide; Th1, type 1 helper T cells; SCVs, *Salmonella*-containing vesicles; TLR, Toll-like receptor.

**Table 2:** The interleukins orchestrate mucosal immune responses to enhance *Salmonella* colitis.

Interleukin	Biologic Functions	Experiments	Intervention and Effects	Clinical Applications	Ref.
IL-1β	Amplifying intestinal inflammation by increasing intestinal epithelial tight junction permeability Atg16L1 suppresses IL-1β expression in macrophage and IECs	IECs	Active vitamin D decrease IL-1β response to *Salmonella* infection to prevent the host from detrimental inflammation	IBD	[2,47,48,49,50]
		Mice	Active vitamin D3 attenuates the severity of *Salmonella* colitis in mice by decreasing IL-1β response		
		Rabbit	Blockade of IL-1 receptors reduces the inflammatory responses in experiment colitis		
IL-8	Accumulation of neutrophils gives rise to colitis and sepsis	IECs	Probiotics suppress IL-8 expression	*Salmonella* colitis IBD	[1,25,51]
			2. Active vitamin D3 suppresses IL-8 expression		
IL-10	Promote systemic *S.* Typhimurium infection in mice	Mice	Anti-IL-10 monoclonal antibody block IL-10 to defense against systemic *Salmonella* infection	*Salmonella* sepsis	[52,53]
IL-12	A pro-inflammatory cytokine in response to microbial pathogens	Humans	Ustekinumab, the monoclonal antibody targeting the shared p40 subunit of IL12/IL23, has been approved for treatment of IBD	IBD	[30,54]
IL-15	Pro-inflammatory by itself Promote intestinal dysbiosis that increases susceptibility to colitis	Mice		Celiac disease IBD	[32,55,56]
IL-17	Orchestrate mucosal inflammation in IBD and *Salmonella* colitis iNKT cells play a protective role against *Salmonella*-enterocolitis by downregulating IL-17-producing γδT cells	Macrophages iNKT cells	*Lactobacillus plantarum* Lp62 was able to suppress IL-17, IL1-*β* and TNF-*α* production in LPS-stimulated J774 macrophages	*Salmonella* colitis IBD	[36,57,58]
IL-18	A member of the IL-1 family of cytokines with pro-inflammatory and tumor-suppressive properties Initiates a pro-inflammatory cytokine cascade in peripheral blood mononuclear cells (PBMC)		*Salmonella* pathogenicity islands (SPI)-1 effector secretion leads to NF-kB signaling and caspase-1-mediated IL-1β/IL-18 activation Animal models suggest suppression of IL-18 bioactivity as a novel therapeutic concept specifically for the treatment of chronic inflammatory diseases		[37,59,60]
IL-23	Accelerate proliferation of both murine and human memory T cells producing Th17 cytokines including iIL-17 and IL-22 Increased production of IL-23 in various mouse models of colitis and IBD patients	Humanmice	Neutralizing antibodies against IL-12/IL-23 p40 and IL-23 p19 have been successfully used in clinical trials for therapy of IBD	IBD *Salmonella* colitis	[41,61,62,63]
IL-27	IL-27 can directly induce expression of IL-1 and TNF-α by primary mast cells and production of IL-1, TNF-α, IL-12p35 and IL-18 by monocytes	Monocytes Mast cells		*Salmonella* infection	[44]

Abbreviations: IECs, intestine epithelial cells; IBD, inflammatory bowel disease; MDP, muramyl dipeptide; NK, natural killer; Treg: regulatory T cells; NKT, natural killer T; INF, interferon; TNF, tumor necrosis factor; LPS, lipopolysaccharide; Th1, type 1 helper T cells; SCVs, *Salmonella*-containing vesicles; TLR, Toll-like receptor.
